# A modified hybrid transport technique combined with a retrograde tibiotalocalcaneal arthrodesis nail for the management of distal tibial periarticular osteomyelitis and associated defects

**DOI:** 10.1186/s13018-023-03744-2

**Published:** 2023-03-30

**Authors:** ChaoFeng Wang, Teng Ma, Zhao Li, Qian Wang, Zhong Li, Kun Zhang, Qiang Huang

**Affiliations:** Department of Orthopedics, Hong Hui Hospital, Xi’an Jiaotong University, Xi’an, 710054 Shaanxi China

**Keywords:** Bone transport, Bone defect, Distal tibia, Tibiotalocalcaneal arthrodesis nail, Calcium sulfate

## Abstract

**Background:**

This paper aimed to propose a modified technique of bone transport. An annular frame combined with a retrograde tibiotalocalcaneal arthrodesis nail was used in this novel technique for treating large distal tibial periarticular osteomyelitis and associated defects.

**Methods:**

Our team conducted a retrospective research. Forty-three patients with large distal tibial periarticular bone loss were involved in this study. Sixteen patients were treated using the modified hybrid transport technique (MHT group) while 27 were subjected to traditional bone transport (BT group). The mean bone loss was 7.8 ± 2.4 cm in the MHT group and 7.6 ± 2.6 cm in the BT group. The external fixation index, time in transport frame, self-rating anxiety scale, bone healing results and postoperative complications were recorded.

**Results:**

The mean time in frame for the MHT group was 3.6 ± 1.5 months, while that of the BT group was 10.3 ± 2.7 months (*p* < 0.05). The mean external fixation index of MHT group was 0.46 ± 0.08 months/cm versus 1.38 ± 0.24 months/cm of the BT group (*p* < 0.05). There was no statistical difference for the bone healing results between the MHT and BT groups (*p* = 0.856). The self-rating anxiety scale and total complication incidence of the MHT group were significantly lower than that of BT patients (*p* < 0.05).

**Conclusion:**

Compared to the traditional BT technique, our modified hybrid transport technique showed better clinical outcomes for treating large distal tibial periarticular bone loss, including less time in transport frame, lower external fixation index and complication incidence. Therefore, this modified technique should be further promoted and developed.

## Background

Patients with a severe open fracture, osteomyelitis or nonunion of the distal tibia are prone to large segmental bone defects [1]. Measures for managing large segmental bone loss comprise bone transport, induced membrane technique and bone grafting with or without a vascular pedicle. [2–5]. Yet, the number of autologous bones available for transplantation is limited. Moreover, wounds will occur in the donor area, and there are possible complications, such as donor site infection and chronic pain. Therefore, these issues limit the wide application of autologous bone transplantation and induced membrane technique. Meanwhile, pedicled fibular transplantation is a good strategy. However, the soft tissue in the fibular flap area needs to be intact. Moreover, the free fibular flap surgery is complicated and risky as it needs vascular anastomosis. Indeed, long periods of fibular hypertrophy can hinder patient ambulation and facilitate fibular fracture [5]. Bone transport is currently the gold standard technique for massive bone loss as it does not need autologous soft tissues and bone grafting. However, one obvious shortcoming of this technique is the verbose time in transport frame, which leads to several complications and patient discomfort [6, 7].

Scholars try to shorten the time in transport frame and introduce several modified techniques, like hybrid transport, shortening and re-lengthening, first bone transport and then nailing, trifocal, tetrafocal and even pentafocal bone transport [8–14]. The technique of hybrid transport was shown to significantly reduce the external fixation index and corresponding complications. Indeed, transport over a nail is superior to that over a plate, as the intramedullary nail belongs to central fixation and allows early weight bearing. Of note, the transport fixator is removed when the transport period is complete. Sen et al. reported cases of bone transport over a femoral retrograde nail for managing infected femur nonunion with bone loss [11]. Several other scholars introduced hybrid transport by using a tibial nail for treating large segmental tibial defects [8, 15, 16]. However, tibial intramedullary nails cannot offer enough stability for patients with distal tibial periarticular bone loss. Therefore, transport over traditional tibial nails is not suitable to patients who suffer distal tibial periarticular bone loss.

**Fig. 1:**
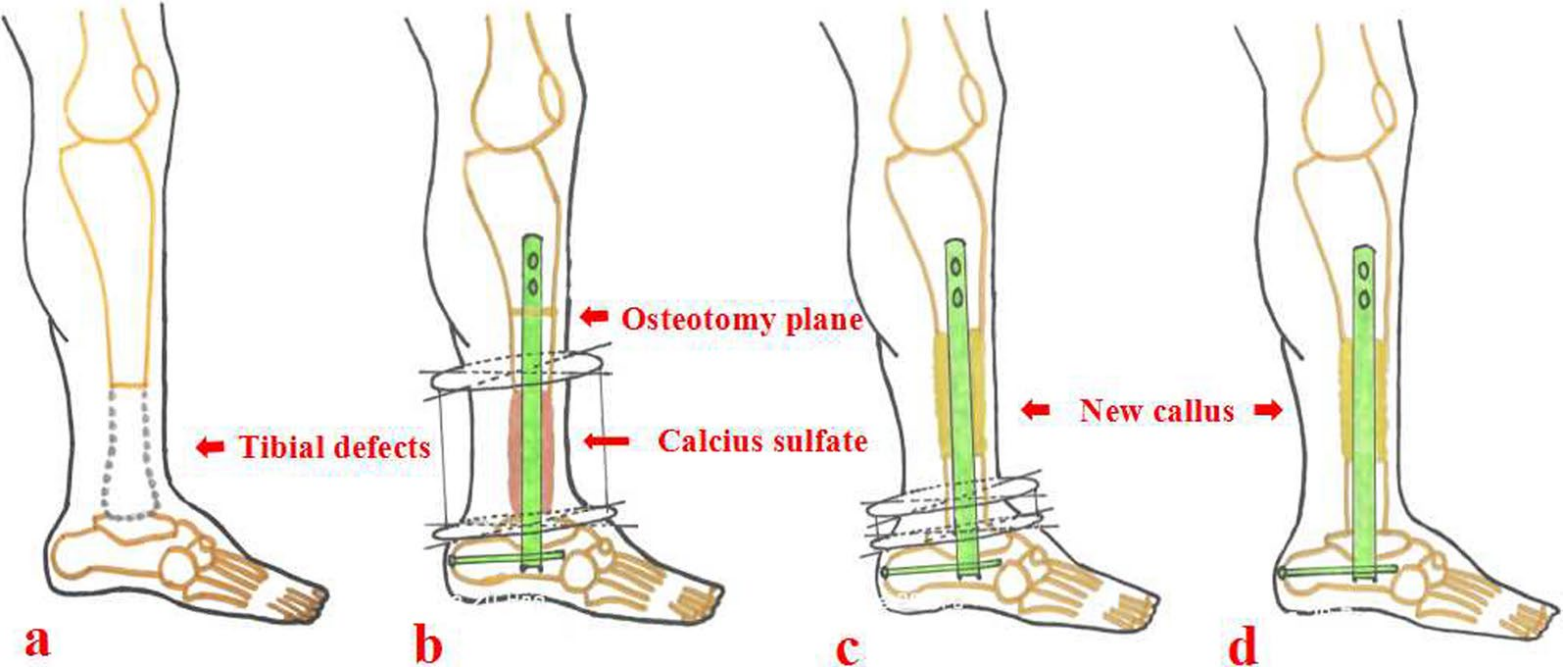
Schematic diagram of the MHT technique. **a** Patient suffering from segmental distal tibial defects; **b** An annular frame combined with a retrograde tibiotalocalcaneal arthrodesis nail is installed and antibiotic-loaded calcium sulfate is filled into the bone defect site; **c** New callus generated after gradual bone transport; **d** After the bone transport period finishes, the transport frame is removed. MHT: modified hybrid transport

The authors have created a modified technique of bone transport (as shown in Fig. 1) for the management of distal tibial periarticular defects, in order to make up the shortage of traditional Ilizarov transport technique. A retrograde tibiotalocalcaneal arthrodesis nail combined with the annular transport frame was used and calcium sulfate spacer was filled in the distal tibial area. We aimed to introduce our experience with this modified hybrid transport technique for treating distal tibial periarticular bone loss.

## Materials and methods

### Patients

The biomedical research ethics committee of Hong Hui hospital, Xi’an Jiaotong University has approved this research (No. 202211010). All methods were performed according to relevant guidelines and regulations. A retrospective study was conducted by our team. The clinical and radiological information of 43 cases with distal tibial periarticular bone loss managed by our team from June 2013 to June 2019 were collected and sorted. The distal tibia was defined as the distal third of the total tibial length. Distal tibial periarticular defects meant bone defects within the distal third of the tibia and the involvement of the ankle joint. The inclusive criteria were as follows: (i) those diagnosed with post-traumatic distal tibial periarticular osteomyelitis, severe infection or those who suffered from segmental distal tibial periarticular defects after radical debridement; (ii) most of the ankle joint surface was defective after radical debridement, and ankle fusion was inevitable; (iii) patients treated by the modified hybrid transport or traditional bone transport technique; (iv) patients with complete medical records. The exclusive criteria were defined as follows: (i) distal tibial periarticular bone loss resulted from tumor or congenital diseases; (ii) those suffered from severe comorbidities and were unable to tolerate anesthesia or surgery; (iii) patients whose limbs were severely injured and amputation was unavoidable; (iv) patients with preservable tibiotalar joint and subtalar joint.

### Preoperative treatment

In order to assess inflammatory indexes, routine blood sampling test was conducted for each patient. The deep secretions of wounds were collected for bacterial culture and detection of antibiotic resistance genes. Radiological filming and three-dimensional reconstruction of distal tibia were conducted before surgery. All patients were subjected to radical debridement in phase one. The chief surgeon removed all sequestrum and completely free fragments. For a radical debridement, segmental resection was commonly used. All the cases had been resected the ankle plafond. When the bone ends showed punctate bleeding, the bone debridement was considered to be sufficient. The vitality of skin and soft tissues was mainly judged based on color, contraction, circulation, and tenacity. The residual tibial defects were filled with bone cement (CORIN Ltd., Cirencester, Gloucestershire, UK). The distal tibia was then fixed with an external fixator temporarily (Naton Medical Instrument Co., Ltd., Tianjin, China). The residual soft tissue defects were treated by skin grafts or flaps according to the “Reconstructive Ladder” principles. It was usually 6–8 weeks between the 1st stage procedure and 2nd stage procedure. Infection controlling and soft tissue conditions were the most important factors in deciding to perform 2nd stage procedure. After the infection was completely controlled and the skin and soft tissues healed, the 2nd stage procedure was performed.

### Surgical procedures

**Fig. 2:**
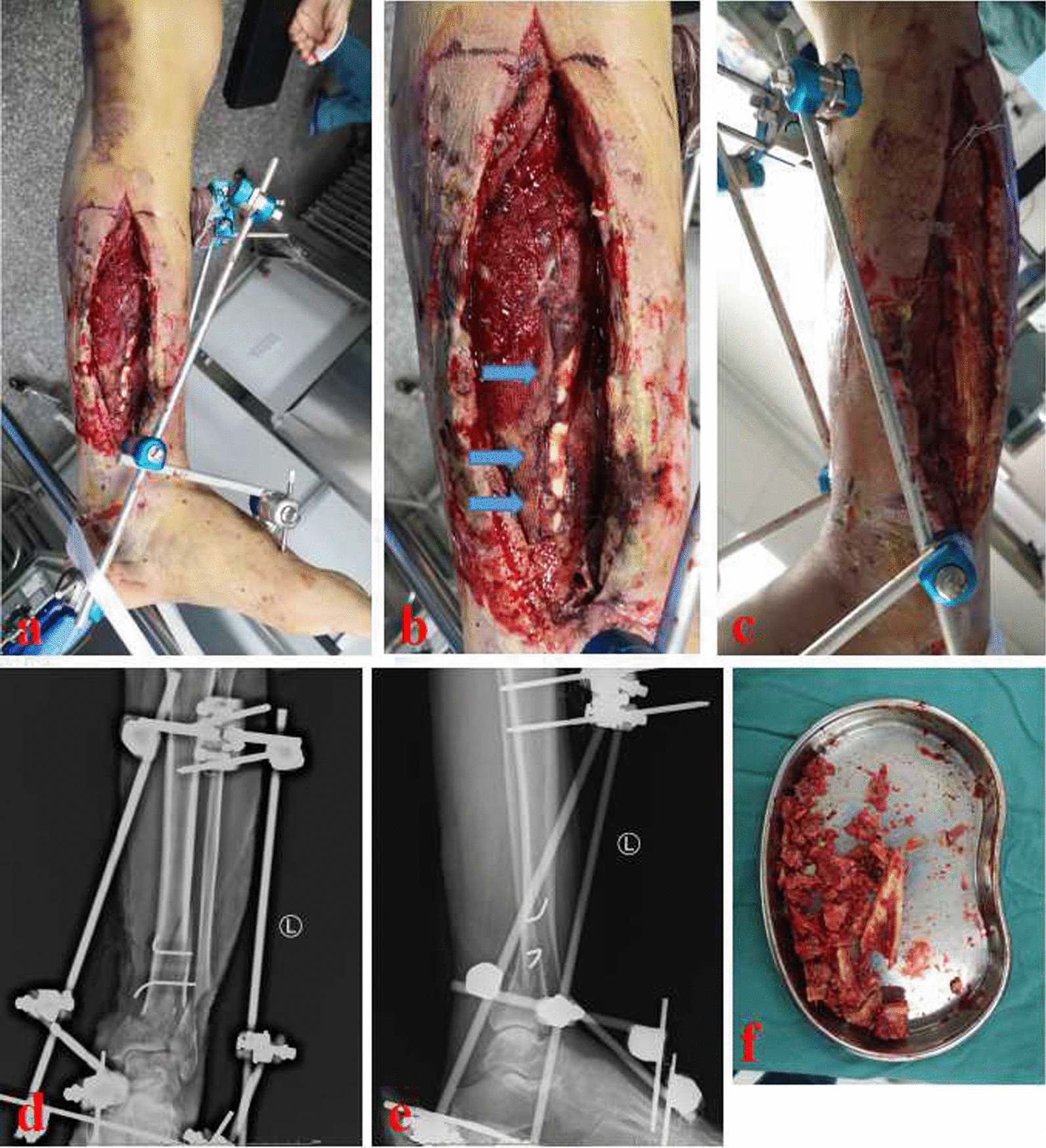
A 51-year-old male suffered from osteofascial compartment syndrome and distal tibial osteomyelitis. **a**–**c** When the patient came to our hospital, the initial appearance of the injured leg was recorded. Blue arrows indicate infected bones at the distal tibia; **d** and **e** Initial X-ray films of the distal tibia; **f** The plate shows sequestrum and infected bone tissues removed during the first debridement in our hospital

**Fig. 3:**
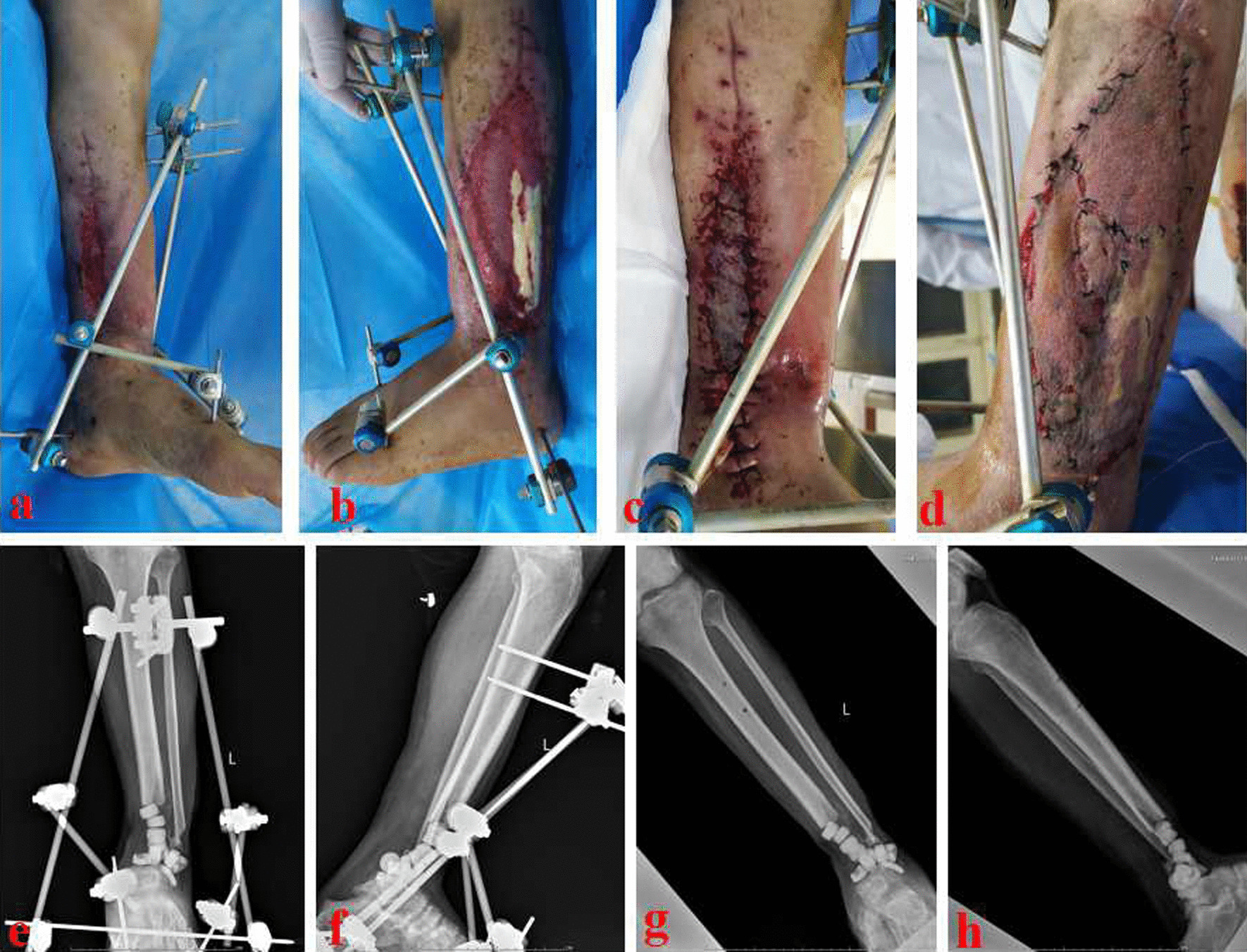
The patient underwent debridement, bone cement filling and soft tissue reconstruction. **a** and **b** After debridement, there was still a large area of skin and soft tissue defects; **c** and **d** The skin and soft tissue defects were repaired by skin grafting;** e** and** f**: X-ray films after bone cement filling; **g** and **h** X-ray films after removing the temporary external fixator

**Fig. 4:**
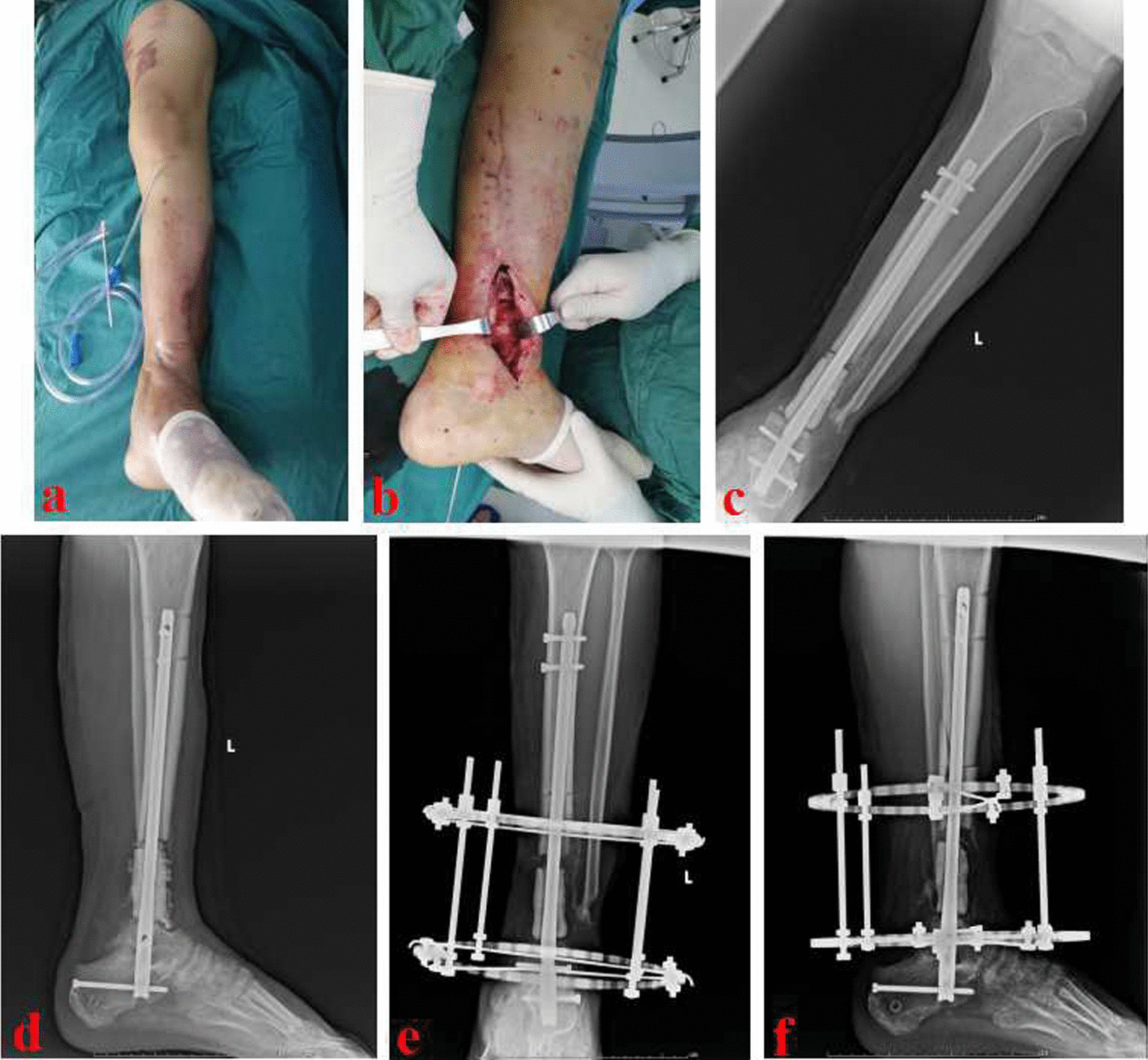
The patient underwent bone reconstruction through the MHT technique. **a** Appearance of the lower leg after wound healing; **b** After removing the bone cement, obvious bone defects (about 8.0 cm) was observed in the wounds; **c** and **d** X-ray films after the insertion of a retrograde tibiotalocalcaneal arthrodesis nail and antibiotic-loaded calcium sulfate; **e** and **f** After two weeks, the annular bone transport frame was installed. MHT: modified hybrid transport

**Fig. 5:**
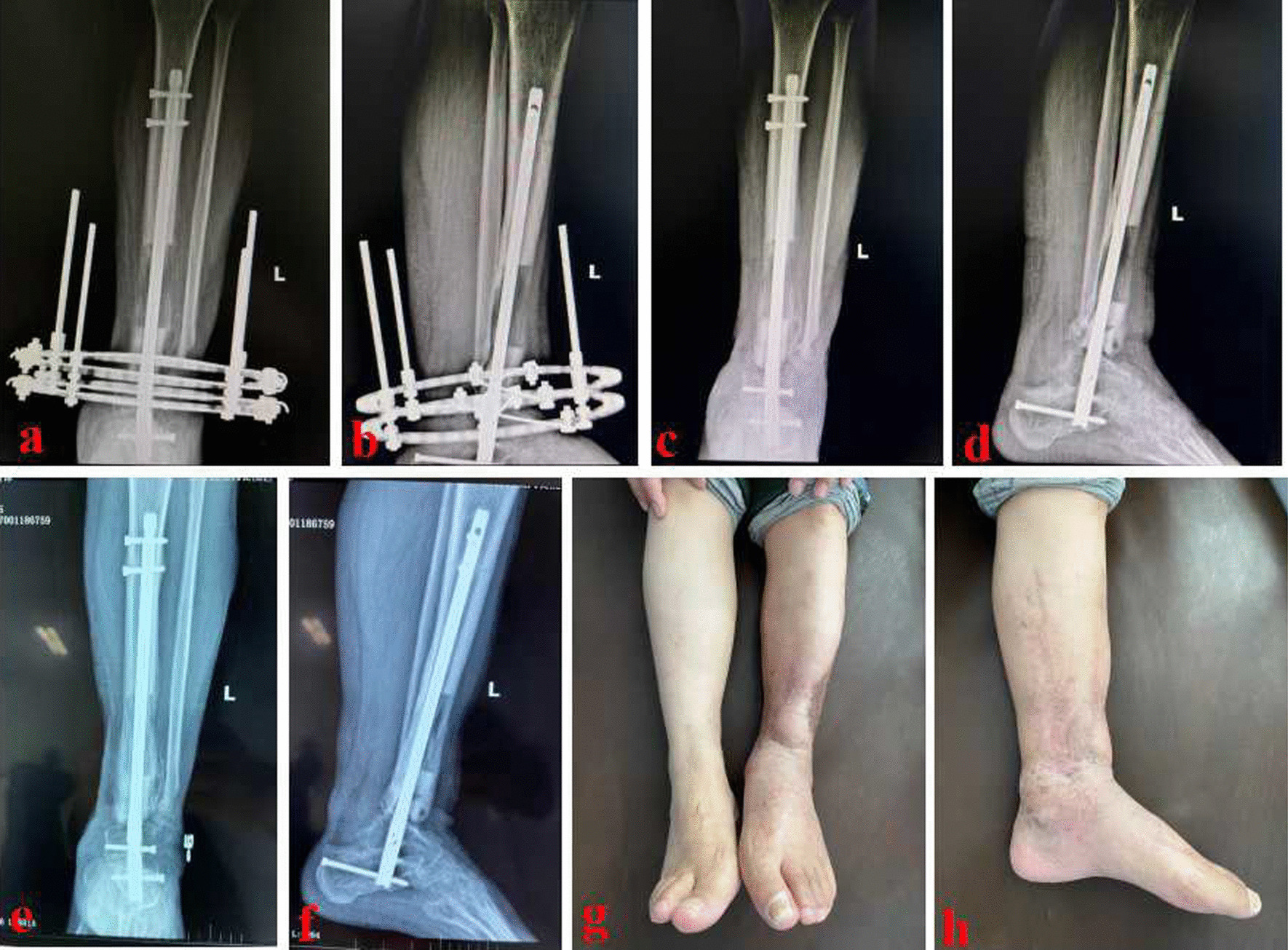
The patient underwent bone transport and was successfully treated using the MHT technique. **a** and **b** After three months, the bone transport period finished and new callus was generated; **c** and **d** X-ray films after removing the annular transport frame; **e** and **f** After six months, X-ray films showed an appropriate consolidation of new callus; g and **h** Appearance after the reconstruction of distal tibial defects. MHT: modified hybrid transport

Surgical procedures of the MHT group were as follows. The temporary external frame was removed first. The former bone cement was exposed and removed. Debridement was performed again at the bone defect site. Then, an antibiotic-loaded calcium sulfate spacer (Biocomposites. Ltd, UK) was prepared. Antibiotics were selected based on drug sensitivity and bacterial culture tests. The antibiotic-loaded calcium sulfate spacer was made into a hollow pipe structure. Later, at the sole of the foot, the entry point of the retrograde tibiotalocalcaneal arthrodesis nail was identified by the image intensifier. The guide pin passed through the calcaneus and talus and entered the medullary cavity of the distal tibia. The medullary cavity was enlarged along the direction of the guide pin. The calcium sulfate spacer was filled into the bone defect site. After that, an appropriate retrograde tibiotalocalcaneal arthrodesis nail (Naton Medical Instrument Co., Ltd., Tianjin, China) was inserted through the center of the calcium sulfate spacer. The axis and rotation of the injured limb were corrected under the image intensifier. Both ends of the tibiotalocalcaneal arthrodesis nail were locked. According to conditions of the surrounding soft tissue, the annular bone transport frame (Naton Medical Instrument Co., Ltd., Tianjin, China) was installed at the same time or two weeks later. When the annular frame was installed, the distal ring was fixed to the talus. Low energy osteotomy was conducted in the proximal tibia. The proximal ring was fixed on the transport fragment. One week after the annular transport fixator was installed, distraction osteogenesis began. The initial transport speed was set at 1.0 mm/day, which was performed in several times. The transport speed was adjusted according to limb tolerance and osteogenesis. After the docking site was in contact, it was pressed properly. Then, the annular transport fixator would be demolished. The arthrodesis procedure was performed in all cases during bone transport process. When the docking site healed, tibiotalar joint fusion was completed at the same time. Figures 2, 3, 4 and 5 show a typical case of the modified hybrid technique.

For the BT group, the transport method was similar to that explained in previous literature [8]. The lower leg was maintained in the center of the transport frame. Parallel to the knee and ankle joint surface, the transport frame was fixed with several Kirschner wires. The proximal rings were fixed at the proximal tibia while the distal rings were fixed at the calcaneus and talus. According to the surgical plan, the proximal tibial osteotomy was taken. Schanz nails were inserted to fix the transport segment. Under a C-arm intensifier, the tibial alignment and rotation were checked. One week after surgery, the distraction osteogenesis process began. Bone transport stopped after the docking site contacted each other. The transport frame would be removed after the consolidation period finished. The bone consolidation time was usually 2–3 times of the bone transport time.

### Postoperative treatment

Erythrocyte sedimentation rate (ESR), C-reactive protein (CRP) and white blood cell (WBC) count were assessed regularly. Systemic antibiotics were usually used for six weeks. To achieve a good limb function, patients were guided to take appropriate exercises and weight bearing after surgery. They were followed once half a month in the first two months and then once every month. Follow-up methods included outpatient, telephone and WeChat platforms. The key points of follow-up included callus formation, complications, etc. The chief surgeon would take timely and effective measures to deal with different complications. Pin-tracts were nursed every day after operation. Both MHT and BT patients began to bear partial weight after the second stage of surgery.

### Outcome measurement

The time in frame, external fixation index and self-rating anxiety scale [17] were recorded during the follow-up period. The time in frame was the total time from installing the transport frame to removing the frame. External fixation index was the value obtained by dividing the time in frame by the length of bone boss. The self-rating anxiety scale was the value evaluated by a subjective test scale one month after the transport frame was installed. It included no anxiety, mild anxiety, moderate anxiety, etc. Bone healing results were calculated and evaluated according to Paley score which included four aspects [18]. That was (i) bone healing, (ii) no infection recurrence, (iii) limb deformity less than seven degrees, and (iv) unequal length of limbs less than 2.5 cm. Bone healing results were classified as excellent, good, fair and poor. Excellent meant good bone healing and (ii)–(iv) indicators for all projects. Good was defined as good bone healing and (ii)–(iv) indicators for two projects. Fair meant good bone healing and (ii)–(iv) indicator for one project while poor stood for bone nonunion or refracture + (ii)–(iv) indicators for no projects. Postoperative complications were classified based on Paley criteria [19], comprising problems, obstacles, and sequelae. A problem is defined as a potential expected difficulty that arises during the distraction or fixation period that is fully resolved by nonoperative means. An obstacle is defined as a potential expected difficulty that is fully resolved by operative means. The total number of cases that had complications was counted and each complication was compared between the two groups.

### Statistical methods

The SPSS 24.0 software (IBM Co., USA) was used to process and analyze data. Measurement data are presented as mean ± standard deviation and were compared using an unpaired t-test, including age, bone loss, body mass index (BMI), ESR, CRP, WBC count, follow-up, time in frame, and external fixation index. Count data were expressed as number or number + incidence and were analyzed using the χ2 test. Count data included gender, classification, bone healing results, self-rating anxiety scale, and complications. *p* < 0.05 was used to infer statistical significance.

## Results

### General data

As shown in Table 1, 43 patients suffering from distal tibial periarticular osteomyelitis and defects were included, comprising 32 males and 11 females. The mean age was 38 ± 12 years in the modified hybrid transport group versus 39 ± 10 in the bone transport group, while the mean bone loss was 7.8 ± 2.4 cm and 7.6 ± 2.6 cm for MHT and BT patients, respectively. Likewise, the mean body mass index was 26.2 ± 2.7 kg/m^2^ and 25.0 ± 3.3 kg/m^2^, for MHT and BT patients, respectively. All cases were categorized based on the Cierny–Mader standard, including medullary (Type I), superficial (Type II), localized (Type III) and diffuse (Type IV) [20]. There were five type III and 11 type IV cases in the MHT group while eight type III and 19 type IV in the BT group. The preoperative erythrocyte sedimentation rate was 16.3 ± 12.8 mm/h for MHT patients versus 15.6 ± 13.5 mm/h for BT ones. There were no differences regarding C-reactive protein, white blood cell count and average follow-up time between groups (*p* > 0.05).

**Table 1:** Comparison of demographic information between the two groups

Variable	MHT group (*n* = 16)	BT group (*n* = 27)	*p* value
Age (year)	38 ± 12	39 ± 10	0.781
Gender (M/F)	12/4	20/7	0.769
Bone loss (cm)	7.8 ± 2.4	7.6 ± 2.6	0.800
Body mass index (kg/m^2^)	26.2 ± 2.7	25.0 ± 3.3	0.204
Cierny–Mader classification			0.819
Type III	5	8	
Type IV	11	19	
ESR (mm/h)	16.3 ± 12.8	15.6 ± 13.5	0.866
CRP (ng/L)	13.35 ± 9.52	14.27 ± 8.85	0.756
WBC count (× 10^9^/L)	10.24 ± 3.36	12.58 ± 4.15	0.051
Follow-up (month)	32 ± 5	33 ± 5	0.531

MHT stands for modified hybrid transport. BT stands for bone transport. ESR stands for erythrocyte sedimentation rate. CRP stands for C-reactive protein. WBC stands for white blood cell

### Comparison of clinical indexes of the two groups

As shown in Table 2, the mean time in frame was 3.6 ± 1.5 months for the MHT group and 10.3 ± 2.7 months for the BT group, respectively (*p* < 0.05). With respect to the external fixation index, it was 0.46 ± 0.08 months/cm for the MHT group and 1.38 ± 0.24 months/cm for the BT group (*p* < 0.05), respectively. The docking site healed for all 43 patients finally. No significant difference was observed in terms of bone healing between the two groups (*p* = 0.856). Specifically, we found ten patients with an excellent recovery, four with a good recovery and two with a fair recovery in the MHT group. For the BT group, 15 patients had an excellent recovery, seven had a good recovery and five had a fair recovery. Results of the self-rating anxiety scale demonstrated that 11 patients from the MHT group did not exhibit anxiety, while five developed mild anxiety. In contrast, three patients from the BT group did not exhibit anxiety, while 14 developed mild and ten developed moderate anxiety. Altogether, the self-rating anxiety scale was lower for patients in MHT group when compared with that of BT patients (*p* < 0.05).

**Table 2:** Comparison of clinical observation indexes between the two groups

Variable	MHT group (*n* = 16)	BT group (*n* = 27)	*p* value
Time in frame (month)	3.6 ± 1.5	10.3 ± 2.7	0.001
External fixation index (months/cm)	0.46 ± 0.08	1.38 ± 0.24	0.001
Bone healing results			0.856
Excellent	10	15	
Good	4	7	
Fair	2	5	
Self-rating anxiety scale			0.001
No anxiety	11	3	
Mild anxiety	5	14	
Moderate anxiety	0	10	

MHT stands for modified hybrid transport. BT stands for bone transport

### Comparison of complications of the two groups

All postoperative complications are documented in Table 3. For the BT group, 15 problems were found, including ten patients with Grade-II pin-tract infection and five with transient loss of knee movement. Oral antibiotics and regular dressing were provided for those with Grade-II pin-tract infection. For those with transient loss of knee movement, they were guided to conduct active functional exercise. Three of them were subjected to manual release under oral painkillers. Besides, twenty-nine obstacles were found in patients from the BT group, comprising 13 patients with Grade-III pin-tract infection, one with infection recurrence, six with docking site nonunion, seven with axial deviation and two with soft tissue invagination. Grade-III pin-tract infections were managed by taking out the involved pins and inserting a new one. The patient that developed infection recurrence was subjected to radical debridement and given antibiotics for six weeks. Those suffered from docking site nonunion were dealt with autogenous bone grafting. Cases with axial deviation were corrected through another surgery. Patients suffered from soft tissue invagination were subjected to surgical releasing. There were three patients that developed sequelae in the BT group, and they refused advice for further treatment.

**Table 3:** Comparison of complications between the two groups

Variable	MHT group (*n* = 16)	BT group (*n* = 27)	*p* value
Problems			
Grade-II pin-tract infection	2 (12.5%)	10 (37.0%)	0.167
Aseptic exudation	6 (37.5%)	0 (0.0%)	0.003
Transient loss of knee movement	1 (6.3%)	5 (18.5%)	0.505
Obstacles			
Grade-III pin-tract infection	1 (6.3%)	13 (48.1%)	0.005
Infection recurrence	0 (0.0%)	1 (3.7%)	–
Docking site nonunion	1 (6.3%)	6 (22.2%)	0.345
Axial deviation	0 (0.0%)	7 (25.9%)	0.072
Soft tissue invagination	0 (0.0%)	2 (7.4%)	–
Sequelae	1 (6.3%)	3 (11.1%)	0.990
Total number of cases that had complications	9 (56.3%)	25 (92.6%)	0.015

MHT stands for modified hybrid transport. BT stands for bone transport

In the MHT group, nine problems occurred, consisting of two cases of Grade-II pin-tract infection, six of aseptic exudation and one of transient loss of knee movement. Regular dressing was performed for those with aseptic exudation. In addition, one patient developed Grade-III pin-tract infection and another one developed docking site nonunion. One patient developed sequelae and rejected further surgeries. The complication treatment for MHT patients was similar to that of BT patients. The total number of cases that had complications in the MHT group was significantly lower than that of BT patients (*p* < 0.05).

## Discussion

Surgical reconstruction of distal tibial periarticular bone loss resulting from severe open fractures and/or chronic osteomyelitis remains a complex challenge for trauma surgeons. Even if they are handled by experienced professionals, numerous complications and dysfunctions may still occur. The main treatment goal is to prevent amputation and regain satisfactory limb functions. Of note, distal tibial defects and ankle stability should be dealt simultaneously. Here we combined a retrograde tibiotalocalcaneal arthrodesis nail with an annular transport frame for the management of distal tibial periarticular bone loss. Our modified hybrid transport technique could handle large tibial bone loss and ankle stability simultaneously. As a newly designed device for ankle arthrodesis, retrograde tibiotalocalcaneal arthrodesis nails have been successfully applied in patients in need of ankle fusion surgeries [21, 22]. The retrograde tibiotalocalcaneal arthrodesis nail has a central fixation and multi-point stability, which can allow earlier weight-bearing. Nwankwo et al. reported a successful case of bone defect treatment using personalized 3D-printed implant combined with a retrograde tibiotalocalcaneal arthrodesis nail [23]. Milos et al. used a retrograde calcaneo-talo-tibial nail, subtalar arthrodesis and fibula transposition in the treatment of traumatic bone and articulatory defect of the distal tibia and talocrural joint [24]. They demonstrated that the calcaneotalo-tibial nail and transposition of the fibula is another feasible and effective option, especially for unreconstructable joint surfaces [24].

Some scholars have adopted the technique of hybrid bone transport to treat segmental bone loss. Lu et al. evaluated results of post-traumatic femoral defects treated by single-armed external fixator over an intramedullary nail. Eight patients were included and the mean external fixator index was 23.88 days/cm [25]. Oh et al. reported that the mean external fixation index was 26 days/cm using hybrid transport for reconstruction of long bone defects in tibia [26]. In Oedekoven’s study, the external fixation index was 19.42 days/cm for the tibial shaft and 15.93 days/cm for the femur by using the hybrid transport technique [27]. We have been working on the hybrid transport technique for nearly ten years. In our previous study, we presented our experience with a new modified technique of shortening and re-lengthening using a monolateral external frame combined with a calcaneal intramedullary nail for the treatment of distal tibial periarticular post-traumatic defects [28]. Our modified shortening and re-lengthening technique reduced the EFI compared to the bone transport technique. The mean external fixation index in this research was 0.46 ± 0.08 months/cm in patient subjected to the modified hybrid transport technique. This was similar to the above studies of bone transport over a tibial or femoral intramedullary nail. When using a retrograde tibiotalocalcaneal arthrodesis nail, the transport frame could be removed early, while this nail offered biomechanical stability in callus consolidation period. After removing the transport frame, patients felt comfortable and highly satisfied. Indeed, we found that the self-rating anxiety scale was significantly lower in the MHT group when compared with that of patients in the BT group. This shows that patients’ anxiety was significantly improved after removing the transport frame.

Several scholars have reported the postoperative complications of hybrid transport technique. Bas et al. included 40 patients who experienced the lower limb reconstruction by hybrid transport technique from 2000 to 2018 and the total incidence of complications was 57.5%. They found that the hybrid transport technique was related to low cost due to the short treatment cycle, low complication incidence, and rapid rehabilitation [29]. Oh et al. included 12 patients using the hybrid transport technique and 3 of them suffered from postoperative complications [26]. Our research also demonstrated that the total number of cases that had complications was significantly lower in the MHT group when compared with that of the BT group. This was likely due to an earlier removal of the transport frame. There were three patients from the MHT group that developed pin-tract infections. Strikingly, 23 patients from the BT group developed pin-tract infections. This difference may due to the significantly different time in transport frame between the two groups. Patients from the MHT group received system antibiotics as well as had local sustained-release antibiotics. Calcium sulfate loaded with antibiotics is an absorbable local antibiotic sustained-release system which has good drug loading characteristics [30]. The benefits of using such system include precise positioning, high local antibiotic concentrations, few side effects and long treatment time [31]. Several scholars have reported that antibiotic-loaded calcium sulfate was efficacious for treating bone and pin-tract infections [32, 33]. In parallel, BT patients were just given systemic antibiotics. Therefore, our MHT was more efficacious for controlling infection, which could also decrease unplanned surgeries due. In Lu’s study, two patients had infection recurrence after adopting hybrid transport technique [25]. Bas et al. reported four patients of 40 suffered from infection recurrence as postoperative complications [29]. Although there was no infection recurrence after operation for patients in the MHT group, it was a major concern when using a modified hybrid transport technique. Our experience showed that all efforts are needed to reduce this complication, including the application of local and systemic antibiotics, local care of every pin-tract, reducing the time in transport frame, timely soft tissue reconstruction, and enhancement of personal physique. Interestingly, soft tissues were prone to embedding into the defect site and the bone defect ends hardened during distraction osteogenesis process for patients subjected to BT. Yet, in the MHT group, calcium sulfate occupied space, thus avoiding soft tissue embedding. Axial deviation was another common complication in patients of the BT group. This may be related to limb weight-bearing and long time in transport frame. As the insertion of a retrograde tibiotalocalcaneal arthrodesis nail limits the transport direction of new callus, axial deviation did not occur in patients subjected to MHT. In addition, transient loss of knee movement was more common in BT patients than that of the MHT patients. This might result from full-ring application at the level close to the knee joint and the long fixation time in transport frame. This problem might be improved in BT patients by using five-eight ring instead of full-ring.

There were several limitations in our research, such as the limited cases and follow-up time. Besides, this study was a case–control retrospective research. Personal preference of trauma surgeons may affect the formulation of surgical plans. However, this deviation does not significantly influence our current conclusions. We will expand the sample size, prolong the follow-up time and set up a prospective randomized controlled study in a future study.

## Conclusion

Compared with the traditional bone transport technique, our modified hybrid transport technique can reduce the time in frame and associated complications. Those suffering from distal tibial periarticular osteomyelitis and bone loss can achieve an improved satisfaction when subjected to the modified technique. Therefore, this technique is a promising method to improve the treatment of patients with distal tibial periarticular osteomyelitis and defects.

## Data Availability

All data analyzed in this study have been provided in the manuscript.

## Abbreviations

MHT: Modified hybrid transport
BT: Bone transport
ESR: Erythrocyte sedimentation rate
CRP: C-reactive protein
WBC: White blood cell
BMI: Body mass index
